# Nurses’ knowledge and its determinants in surgical site infection prevention: A comprehensive systematic review and meta-analysis

**DOI:** 10.1371/journal.pone.0317887

**Published:** 2025-01-29

**Authors:** Tesfaye Engdaw Habtie, Sefineh Fenta Feleke, Aregash Birhan Terefe, Addis Wondmagegn Alamaw, Melsew Dagne Abate

**Affiliations:** 1 Department of Nursing, College of Health Sciences, Woldia University, Woldia, Ethiopia; 2 Department of Public Health, College of Health Sciences, Woldia University, Woldia, Ethiopia; 3 Department of Emergency and Critical Care Nursing, College of Health Sciences, Woldia University, Woldia, Ethiopia; 4 Department of Nursing, College of Health Sciences, Injibara University, Injibara, Ethiopia; University of Health and Allied Sciences, GHANA

## Abstract

**Objective:**

The objective of this systematic review and meta-analysis is to assess and synthesize the global evidence on the level of nurses’ knowledge and its determinants regarding the prevention of surgical site infections.

**Methods:**

This systematic review and meta-analysis were conducted following strict methodological guidelines to ensure accuracy and reliability. Adhering to the 2020 PRISMA checklist, a systematic review and meta-analysis sought to establish the pooled proportion of nurse’s knowledge and its determinants regarding surgical site infection prevention globally. MeSH terms and keywords were included in the search. Data extraction, quality assessment, and analysis followed established protocols. Heterogeneity and publication bias was assessed using STATA version 17.0.

**Results:**

A total of seventeen observational studies, with sample sizes ranging from 30 to 515 participants, were included in the final analysis in a global context. In this systematic review and meta-analysis, the pooled proportion of nurses with good knowledge of surgical site infection prevention is 62% (95% CI: 50–74%) when assessed using a dichotomous scale. However, when knowledge is measured using a three-point Likert scale, the pooled proportion of those with good knowledge drops to 46% (95% CI: 21–72%), with an additional 27% (95% CI: 16–38%) demonstrating fair or moderate knowledge.

**Conclusion and recommendation:**

This systematic review and meta-analysis is the first to synthesize data on nurses’ knowledge of surgical site infection (SSI) prevention. The findings reveal poor knowledge levels, highlighting the need for targeted educational interventions globally. While the pooled odds ratio is not statistically significant, training, longer service years, and higher education improve SSI prevention knowledge by enhancing critical thinking, boosting confidence, and fostering adherence to evidence-based practices. Future research should focus on identifying factors influencing nurses’ knowledge, particularly through longitudinal and interventional studies. Policymakers should incorporate international guidelines such as those recommended by the World Health Organization (WHO) and the Centers for Disease Control and Prevention (CDC) into nursing curricula, supported by robust assessment tools and educator training, to improve knowledge transfer and implementation of best practices.

## Introduction

Every year, more than 312 million surgeries are performed globally [1]. General surgical procedures are the most frequently performed, especially in high- and middle-income countries [2]. Thus, millions of individuals are at risk for complications resulting from surgery if correct actions and prevention strategies are not applied at appropriate times [3]. ‘Surgical site infection’ (SSI) which was previously known as ‘surgical wound infection’ is a leading cause of healthcare associated infections [3]. In 1992, during the development of guidelines by the CDC, the term ‘surgical site infection’ became the official terminology [4]. SSI is defined as infections occurring within 30 days of a surgical procedure, or within one year if an implant remains in place [4].

Surgical site infections (SSI) can develop either near the surgical incision or within the deeper tissues at the operation site, typically caused by bacteria entering through the surgical cuts. This results in local signs and symptoms such as heat, redness, pain, and swelling [4]. The Centers for Disease Control and Prevention (CDC) categorizes SSIs into three types: superficial incisional (affecting only the skin and subcutaneous tissue), deep incisional (involving deeper soft tissues), and organ/space (affecting any part of the anatomy beyond the incised body wall layers that were opened or manipulated during surgery) [4].

While there have been advancements in surgical procedures, wound care, and sterilization methods, surgical site infections (SSIs) continue to be a common yet preventable complication [5]. A global cumulative incidence of SSIs in general surgery was reported to be 11% in a 2021 study analyzing 57 studies with 488,594 patients [6]. In contrast, a more recent systematic review and meta-analysis conducted in 2023 found a global SSI incidence of 2.5%, though estimates vary across WHO regions. The highest incidence was observed in the African region, with a rate of 2.7% [7]. The overall global incidence of SSIs following appendectomy is 7%, with rates varying from 5.8% in Europe to 12.6% in Africa [8]. In sub-Saharan Africa, the pooled incidence of SSIs is 14.8% [9]. In Nigeria, the pooled cumulative incidence is 14.5% [10], while the pooled prevalence is 5.6% in India [11], 7.9% in the Eastern Mediterranean region [12], and 12.3% in Ethiopia [13].

The high global incidence of SSIs significantly impacts hospital resources and treatment costs, creating a substantial economic burden. On average, SSIs extend hospital stays by 9.7 days and increase costs by $20,842 per admission. Additionally, SSIs lead to readmissions, requiring extra days of care and incurring further costs [14]. This situation also increases the demand for human resources, compromising the care of other patients due to the heightened burden. The impact extends to caregivers, affecting their work time, social interactions, and reducing overall productivity. To reduce the incidence of SSIs and their burden on healthcare systems, the WHO developed new guidelines in 2016. The developed guidelines were based on 30 meta-analyses and systematic reviews by international experts, following WHO’s Guideline Development Process. These guidelines aim to standardize practices before, during, and after surgical procedures [15].

In addition to WHO guidelines, a three-step model has been proposed to prevent surgical site infections (SSIs). This model integrates perioperative measures, multidisciplinary collaboration, and continuous quality improvement (CQI) initiatives [16]. Perioperative measures include identifying patient risk factors, administering appropriate antimicrobial prophylaxis, and ensuring proper skin preparation. Intraoperative measures involve adherence to strict aseptic techniques, wearing appropriate surgical attire, using sterile surgical drapes, and performing antiseptic irrigation. Postoperative measures focus on proper wound care techniques, encouraging early mobilization, and the rational use of antibiotics [16]. Nurses play an invaluable role in these three areas, which are crucial pillars for the prevention of SSIs [16]. The aim of this systematic review and meta-analysis is to identify the level of Nurses knowledge and associated factors towards prevention of SSI globally.

### Research objective and questions

The objective of this systematic review and meta-analysis is to assess and synthesize the global evidence on the level of nurses’ knowledge and its determinants regarding the prevention of surgical site infections (SSIs). What is the pooled proportion of nurses’ knowledge towards the prevention of surgical site infections and what are the key determinants?

## Methods

This systematic review and meta-analysis were conducted following rigorous methodological guidelines to ensure accuracy and reliability. Initially, a comprehensive search of the PROSPERO database was performed to verify the absence of any previously published or ongoing systematic reviews on the same topic. Upon confirming no such reviews existed, the study protocol was submitted to PROSPERO and registered under the number CRD42024581565.

The synthesis of data from eligible primary studies (PS) was conducted meticulously, ensuring that each report was carefully reviewed and integrated into the final analysis. The entire process adhered to established standards for systematic reviews to guarantee a thorough and unbiased synthesis of the evidence ((((Surgical Site Infection) OR (Surgical wound infection)) AND (Nurses Knowledge)) AND (Associated factors).

### Searching strategy and information sources

The authors conducted a comprehensive literature search across multiple electronic databases, including PubMed, Web of Science, Google Scholar, and the Cochrane Central Register of Controlled Trials, to identify studies reporting on nurses’ knowledge and practice patterns regarding the prevention of surgical site infections (SSIs) globally. The search strategy was meticulously designed, combining relevant keywords with Medical Subject Headings (MeSH) terms. It encompassed all articles published up until June 2024, with no restrictions on publication dates to ensure a broad inclusion of literature.

The search strategies were developed using Boolean operators to optimize the retrieval of relevant studies. Keywords and MeSH terms were employed independently and in combination, utilizing "AND" and "OR" to tailor the search for the advanced features of the PubMed database. Additionally, a snowball search of reference lists from retrieved articles was conducted to identify further relevant studies.

Two independent reviewers conducted the literature search, and any discrepancies were resolved through consensus. For articles with incomplete data, corresponding authors were contacted to obtain missing information. The primary search terms included ((((Surgical Site Infection) OR (Surgical wound infection)) AND (Nurses Knowledge)) AND (Associated factors). These terms were applied in various combinations to ensure a thorough search. Following the initial search, full-text articles were reviewed to determine their eligibility for inclusion in the study.

### Study selection

Two investigators, TE and SF, independently conducted the initial screening of studies based on titles and abstracts. This preliminary step was crucial in identifying potentially relevant studies before proceeding to full-text retrieval. In cases where additional information was required to assess the eligibility of a study, the investigators reached out to the corresponding authors for clarification. Any discrepancies between the investigators during the screening process were resolved through thorough discussion, ensuring a consensus was reached on the inclusion or exclusion of studies.

### Eligibility criteria

The retrieved primary studies were exported to Endnote version 7.0 to remove duplicates. We then globally reviewed published primary studies that met the following inclusion criteria: (a) the study specifically addressed the level of nurses’ knowledge and its determinants in relation to SSI prevention. Studies were excluded based on the following criteria: unclear measurement levels, outcomes not clearly stated, unsound methodologies, publications in languages other than English, JBI critical appraisal score ≤3, and studies that did not provide sufficient information for analysis.

### Data extraction

Data were extracted using a structured extraction form by two independent reviewers. In cases where discrepancies in the extracted data were observed, the extraction process was repeated to ensure accuracy. If differences persisted, a third reviewer was consulted to resolve the issue.

### Methodological quality (Risk of bias assessment)

Methodological quality of all included studies were assessed by two independent reviewers using the Assessment of Critical Appraisal tools for use in JBI Systematic Reviews [17]. The quality scoring was done out of 8 points, and it was found to range from 5 to 8,with a mean score of 6.53 points, indicating an overall good quality (**Table 1**).

**Table 1:** Baseline characteristics of studies included nurses’ knowledge about SSI prevention.

Author	Year	Country	Study Design	Sampling technique	Sample S	Level of Good Knowledge % (95% CI)	(JBI Quality indicator
Abd Elhay HA et al.	2016	Egypt	Descriptive research design	convenience sampling	30	0.87(0.74,1.00)	5
Ayamba EVE et al.	2022	Cameroon	Cross-sectional study	Purposive sampling	40	0.65(0.47,0.83)	5
Jaleta P et al.	2021	Ethiopia	Cross-sectional	purposive sampling	218	0.48(0.39,0.58)	8
Haleema Sadia et al.	2017	Pakistan	descriptive correlation study design	Convenient sampling	131	0.36(0.22,0.49)	5
Shaheen SR and Hawash MAH	2021	Egypt	Descriptive research design	convenience sampling	40	0.57(0.37,0.78)	8
Sham F et al.	2021	Malaysia	Cross-sectional	not stated	306	0.85(0.81,90)	6
Woldegioris T et al.	2019	Ethiopia	Cross-sectional	systematic random sampling	208	0.76(0.69,0.82)	8
Teshager FA et al.	2015	Ethiopia	Cross-sectional	Simple random sampling	423	0.41(0.33,0.48)	7
KHALID N et al.	2023	Pakistan	Cross-sectional	Convenience sampling	150	0.75(0.67,0.83)	6
Tiwari RV and Tiwari HD	2022	India	Cross-sectional	simple random sampling	515	0.49(0.43,0.55)	8
Hassan AH and Masror-Roudsary D	2023	Iraq	Cross-sectional	Convenience sampling	180	0.20(0.07,0.33)	6
Naji Msc BA et al.	2020	Baghdad	Cross- sectional	purposive sample	50	0.44(0.23,0.65)	5
Patil VB et al.	2018	India	Cross sectional	Not stated	31	0.97(0.91,1.03)	5
Asmaa Salah EL-Azab1et al.	2023	Egypt	Cross-sectional	Convenience sampling	77	0.20(-0.01,0.40)	8
Mohammed Alsaadi and Elfeshawy R	2024	Iraq	Cross-sectional	Not stated	50	0.88(0.78,98)	5
Joshi R	2014	India	Descriptive study	not stated	120	0.20(0.04,0.36)	5
Famakinwa T et al.	2014	Nigeria	Cross-sectional	Purposive sampling	100	0.32(0.16,0.48)	6

### Statistical analysis

After extracting the data using Microsoft Excel, we imported it into STATA version 17.0 for further analysis. We employed both narrative and qualitative methods to summarize the findings of the included studies. When multiple estimates on the same topic were available, we presented the range of these estimates and calculated a pooled estimate. The standard error for each study was computed using the binomial distribution formula.

We then pooled the overall knowledge and practice rate among nurses, presenting the pooled estimate along with a 95% confidence interval (CI) through forest plots. To assess heterogeneity among the studies, we utilized inverse variance (I²) and p-values. I² values, which range from 0 to 100%, indicate the proportion of the total variance attributable to heterogeneity, with values below 50% suggesting non-significant heterogeneity. Additionally, we calculated a 95% prediction interval (PI) for the summary hazard ratio (SHR), which predicts the true effect size in future studies with 95% certainty, further highlighting the heterogeneity across studies. Sensitivity analysis was conducted to evaluate the impact of individual studies on the overall estimate. Publication bias was assessed visually with a funnel plot and more rigorously using Egger’s and Begg’s regression test.

## Result

**Fig 1:**
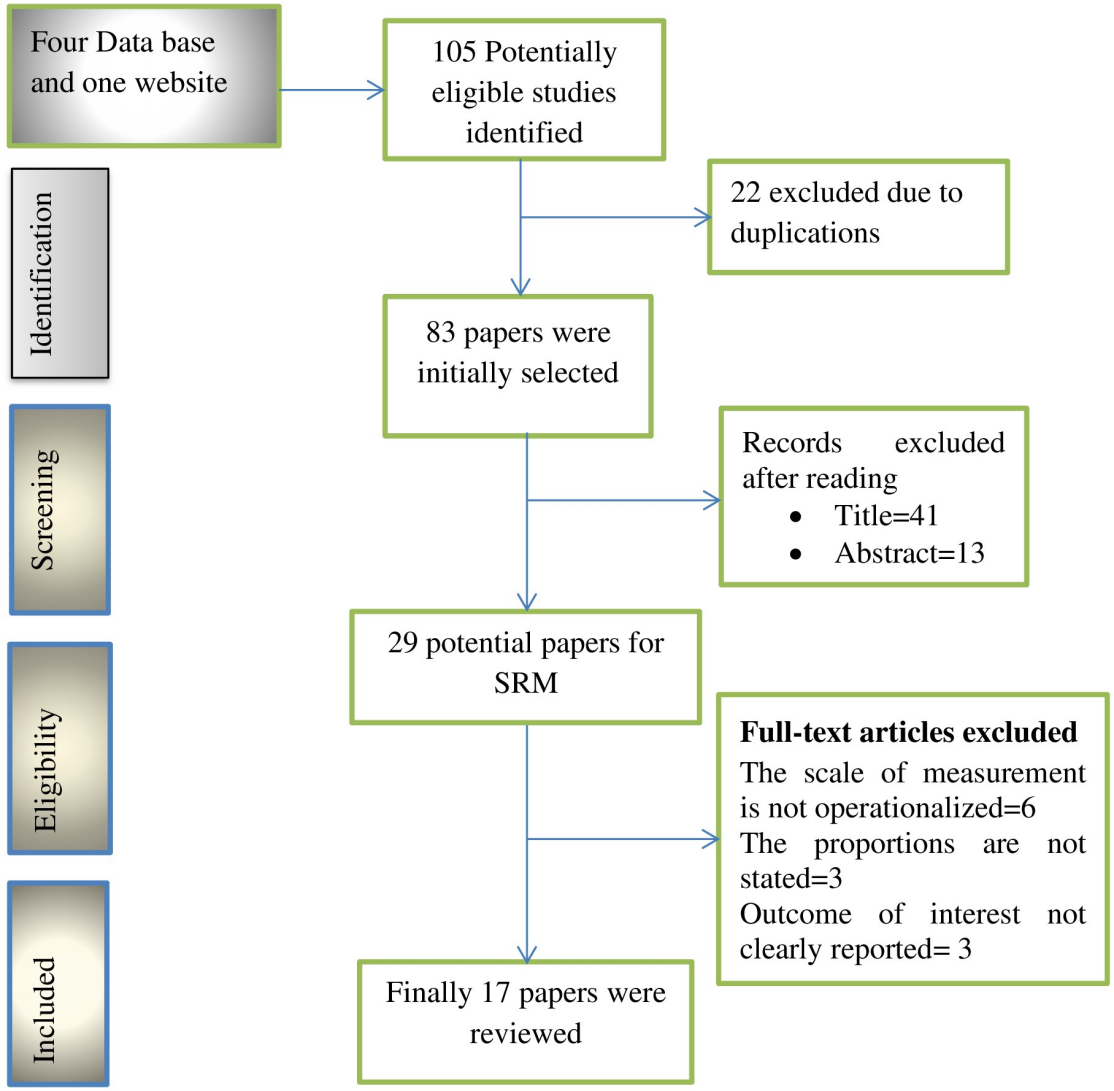
PRISMA flow diagram to illustrate the study selection process.

This systematic review and meta-analysis followed the PRISMA guidelines. The authors searched PubMed, Web of Science, Cochrane, and Google Scholar, identifying 105 papers related to nurses’ knowledge regarding to SSI prevention and its associated factors. After removing duplicates, 83 papers were screened. Of these, 41 papers were excluded based on their titles, and an additional 13 were discarded after abstract review. Ultimately, 17 studies met the inclusion criteria and were included in the final analysis (**Fig 1**).

### Characteristics of included studies

All the papers included in this systematic review and meta-analysis were observational studies published between 2010 and 2024, with sample sizes ranging from 30 to 515 participants. Each of these studies reported on nurses’ knowledge regarding SSI prevention. The following studies specifically reported nurses’ knowledge by categorizing them into good and poor domains: Abd Elhay HA et al. [18], Ayamba EVE et al. [19], Jaleta P et al. [20], Haleema Sadia et al. [21], Shaheen SR and Hawash MAH [22], Sham F et al. [23], Woldegioris T et al. [24], Teshager FA et al. [25], Tiwari RV and Tiwari HD [26], and KHALID N et al. [27].

The following studies were categorized into good, moderate, and poor domains:: Hassan AH and Masror-Roudsary D [28], Naji Msc BA et al. [29], Patil VB et al. [30], Asmaa Salah EL-Azab et al. [31], Mohammed Alsaadi and Elfeshawy R [32], Joshi R [33] and Famakinwa T et al. [34] (Table 1).

### Assessment of publication bias and heterogeneity

**Fig 2:**
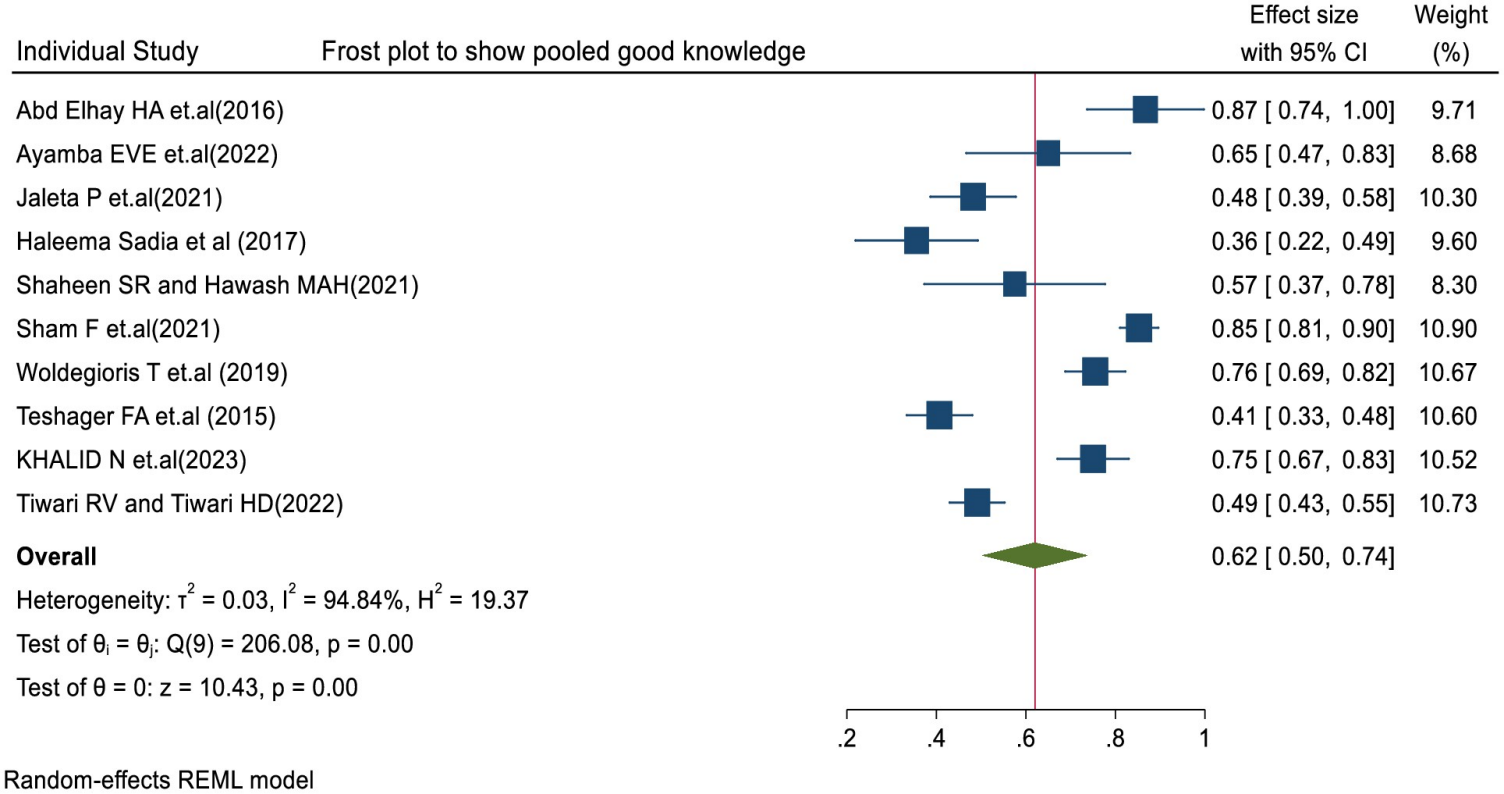
Forest plot of nurses’ good knowledge levels towards SSI prevention.

We also assessed heterogeneity among the included studies using Cochrane’s Q test and the I² statistic. The random-effects model indicated substantial heterogeneity with the value of (I² = 94.84%; p < 0.001) and (Q(9) = 206.08, p < 0.001) (**Fig 2**).

**Fig 3:**
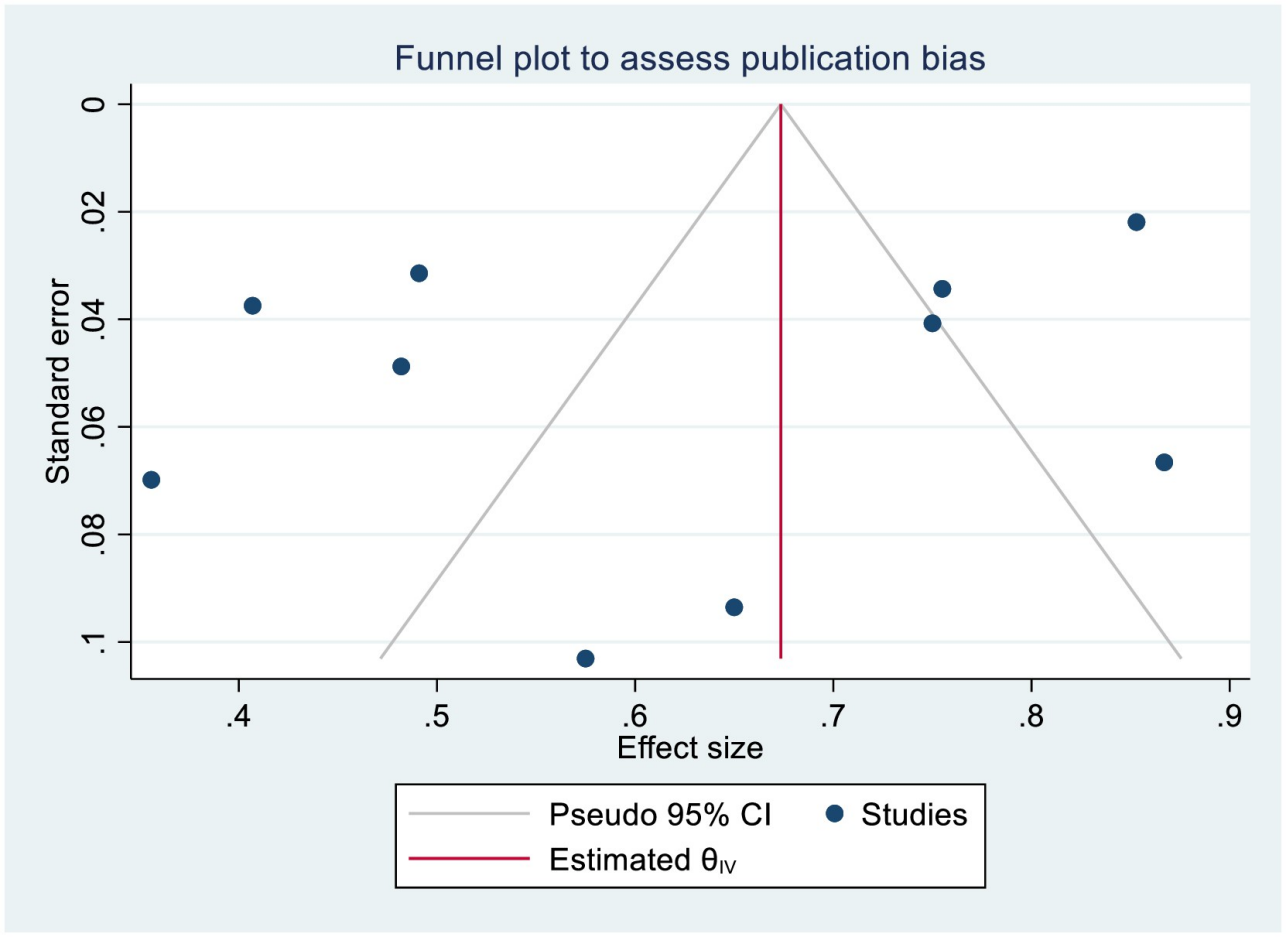
Meta funnels presentation of level of nurses’ knowledge on SSI prevention.

A funnel plot revealed an asymmetrical distribution of the included studies, suggesting the potential for publication bias (**Fig 3**). Recognizing the subjective nature of visual inspection, we conducted more advanced and sensitive statistical tests. Egger’s and Begg’s regression test yielded a coefficient (β) with a standard error (SE) of 2.456, 11.180 and a p-value of 0.6551, 0.7205 respectively indicate that the statistical evidence for publication bias is not strong despite the asymmetrical appearance of the funnel plot. This might be related to asymmetry was mild. To further investigate the presence or absence of publication bias, a trim-and-fill analysis was conducted using a random effects model, but no imputed studies were added. Additionally, a leave-one-out sensitivity analysis was performed to assess the impact of individual studies on the pooled estimate of knowledge level among nurses towards SSI prevention. The sensitivity analysis showed that the pooled findings were robust, as no single study significantly affected the results.

### Pooled knowledge of nurses towards SSI prevention

This systematic review and meta-analysis shows the **Pooled good Knowledge of Nurses towards SSI prevention** globally is 62% (95% CI: 50–74%, p = 0.00).

### Subgroup analysis by level of knowledge

#### Assessment of publication bias and heterogeneity

**Fig 4:**
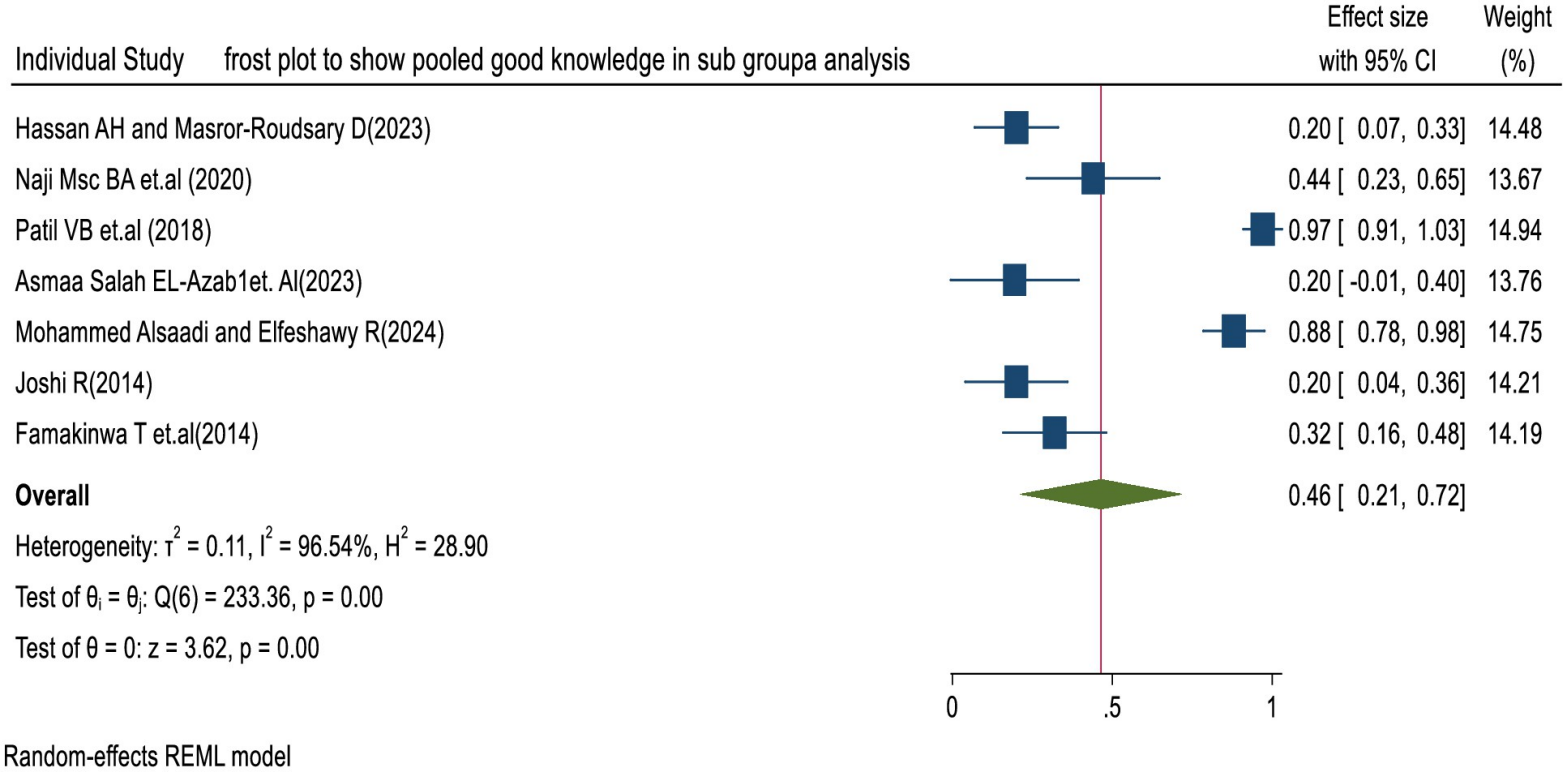
Forest plot of nurses’ good knowledge levels towards SSI prevention in sub group analysis.

We have assessed heterogeneity among the included studies using Cochrane’s Q test and the I² statistic. The random-effects model indicated substantial heterogeneity with the value of (I² = 96.54%; p < 0.001) and (Q(6) = 233.36, p < 0.001) (**Fig 4**).

**Fig 5:**
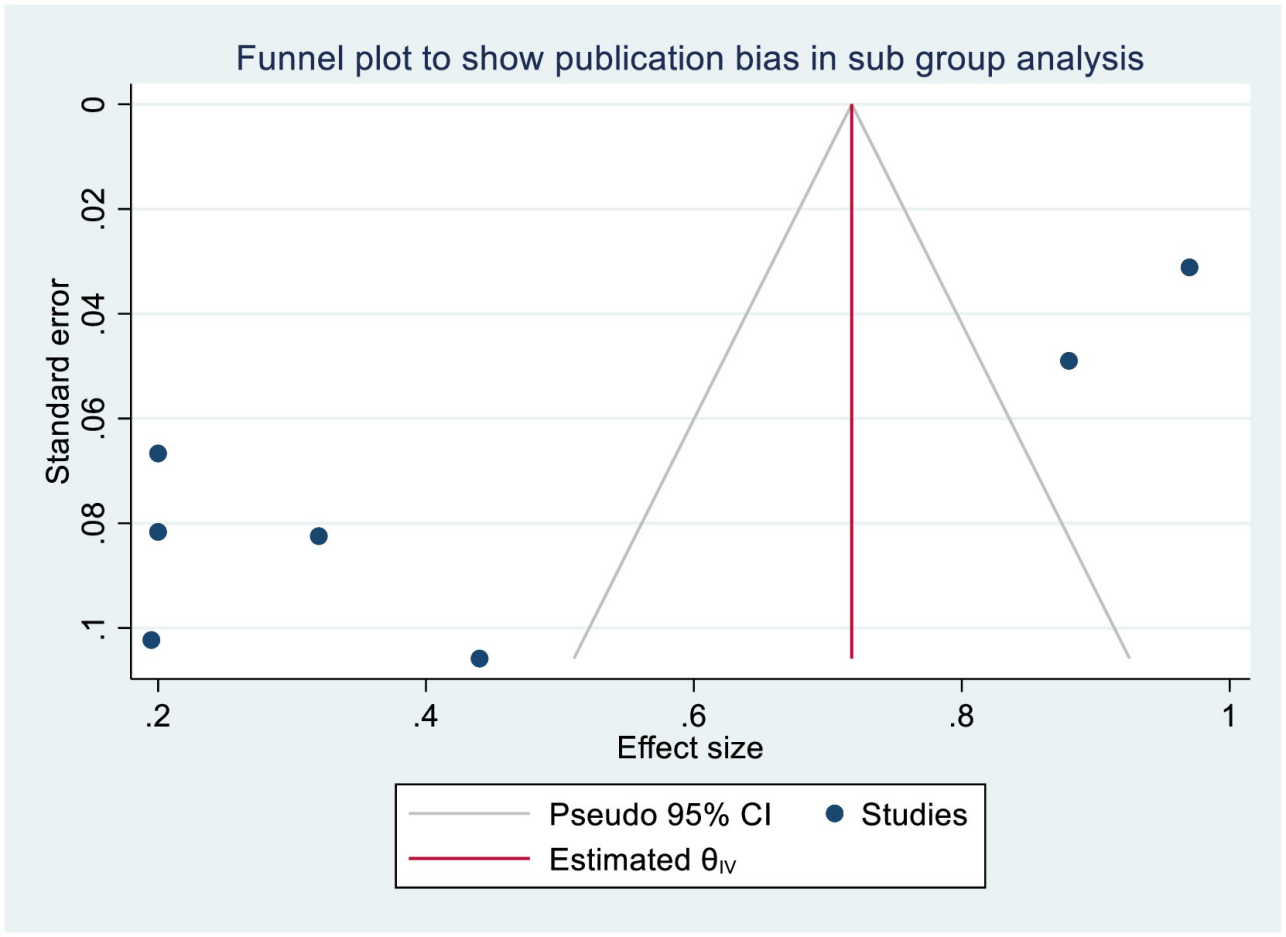
Funnel plot assessing publication bias in studies on nurses’ good knowledge of SSI prevention.

A funnel plot showed an asymmetrical distribution of the included studies suggests the potential for publication bias (**Fig 5**). Egger’s regression test yielded a coefficient (β) with a standard error (SE) of 3.455 and a p-value of 0.0050 indicates that the statistical evidence for publication bias is strong. To further explore the presence or absence of potential publication bias, a trim-and-fill analysis-an important method in meta-analysis used to assess and adjust for such bias-was conducted using a random-effects model. The results indicated that no studies needed to be imputed, suggesting that the asymmetry observed in the funnel plot was minimal and unlikely to have significantly influenced the pooled effect size. Thus, the likelihood of substantial publication bias in this meta-analysis is low. Additionally, a leave-one-out sensitivity analysis was performed to assess the impact of individual studies on the pooled estimate of knowledge level among nurses towards SSI prevention. The sensitivity analysis showed that the result of the pooled findings were affected notably by studies Patil VB et al. [30], Mohammed Alsaadi and Elfeshawy R [32].

#### Assessment of publication bias and heterogeneity

**Fig 6:**
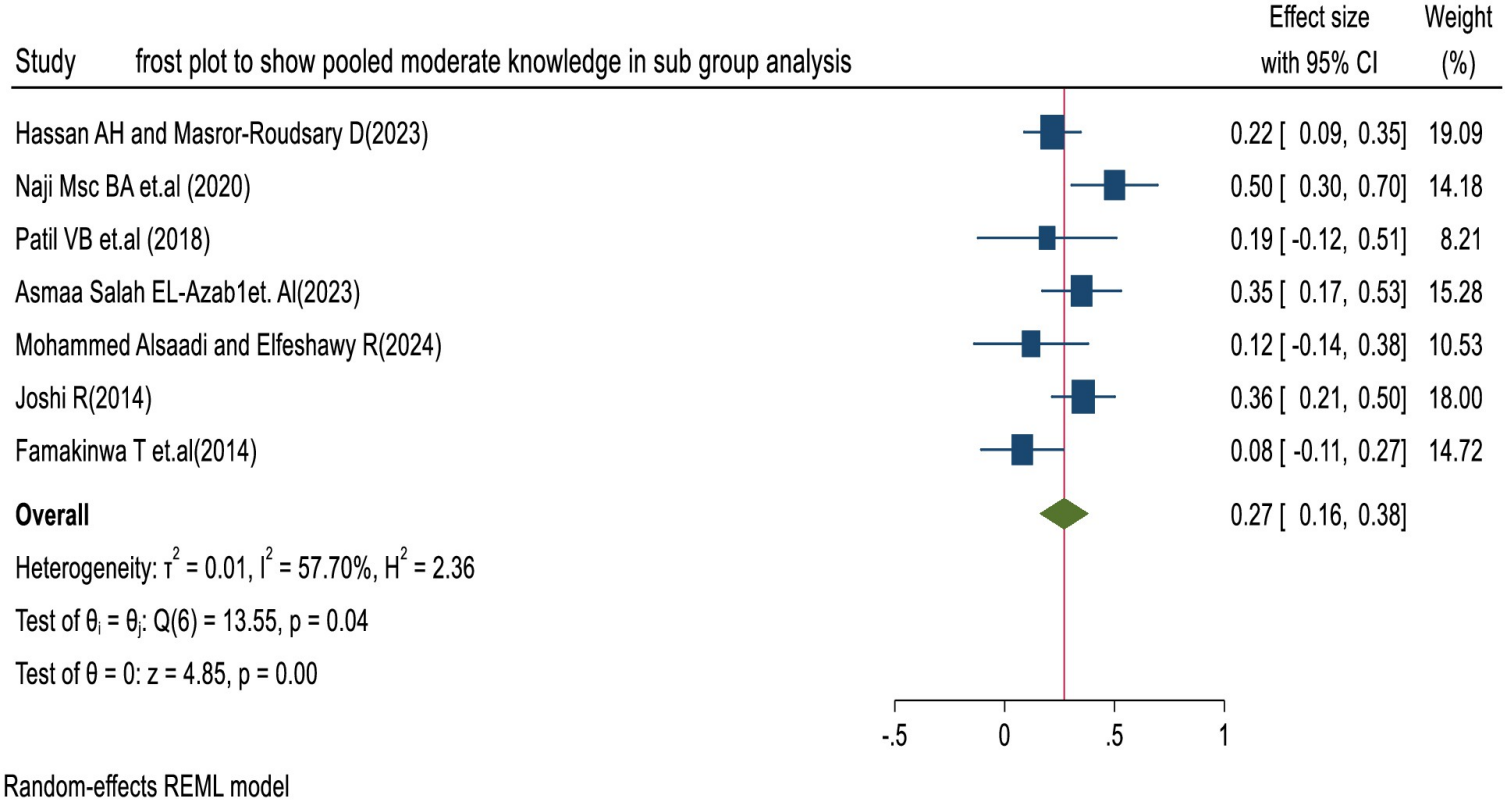
Forest plot of nurses’ moderate knowledge levels towards SSI prevention in sub group analysis.

We assessed heterogeneity among the included studies using Cochrane’s Q test and the I² statistic. The random-effects model indicated moderate heterogeneity (I² = 57.70%; p < 0.001). Cochrane’s Q test showed moderate heterogeneity among the studies (Q(6) = 13.55, p < 0.001) (**Fig 6**).

**Fig 7:**
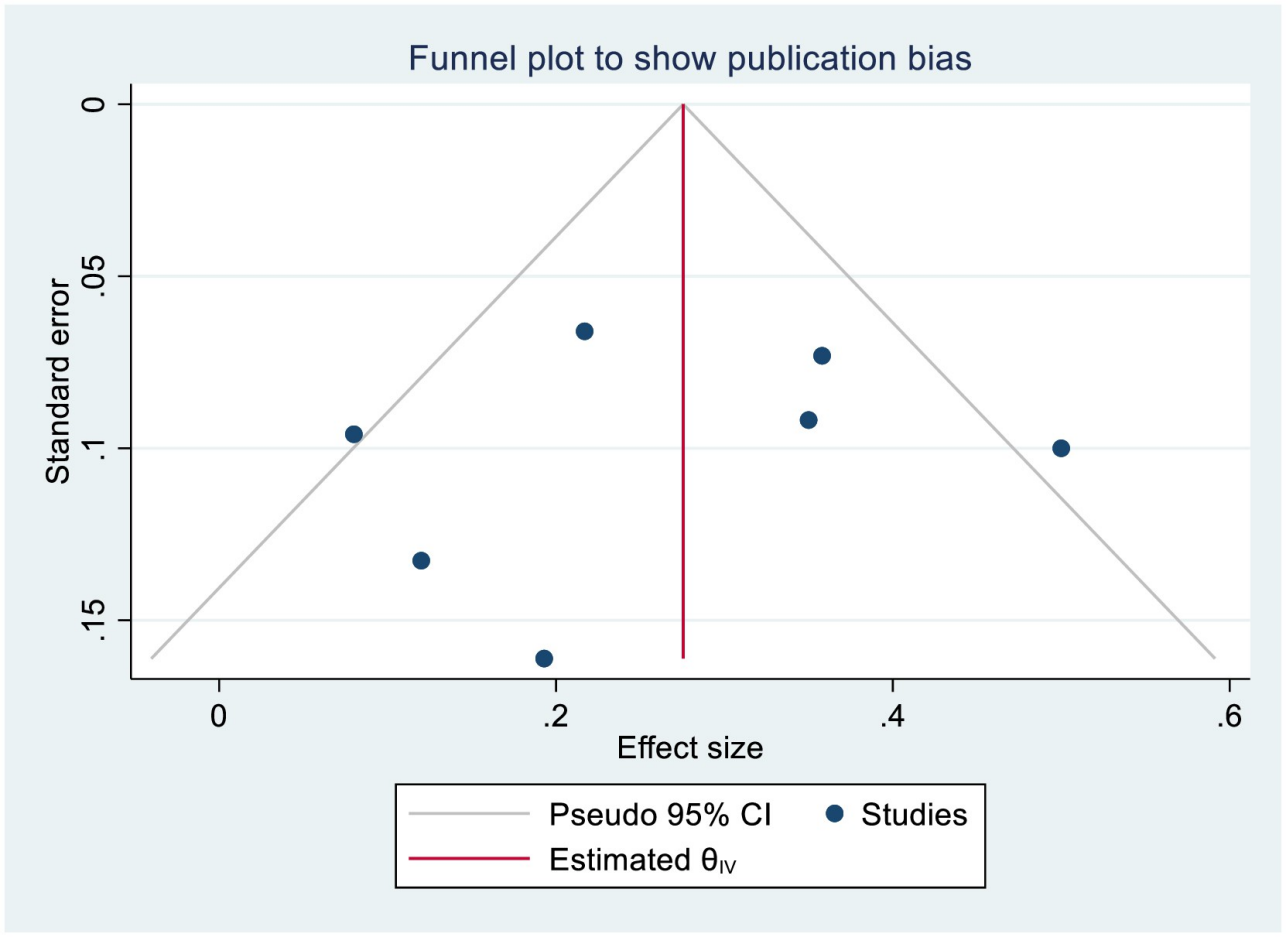
Funnel plot presentation assessing publication bias nurses’ moderate knowledge of SSI prevention.

A funnel plot showed symmetrical distribution of the included studies, suggesting no potential publication bias (**Fig 7**). This finding is supported by Egger’s regression test, which yielded a p-value of 0.5408, indicating there is no statistical evidence for publication bias.

#### Pooled knowledge of nurses towards SSI prevention in sub group analysis

We conducted a subgroup analysis by the scale of measurement to reduce heterogeneity and increase the reliability of the results. The findings of this systematic review and meta-analysis using a three-point Likert scale indicate that the pooled prevalence of good knowledge among nurses towards SSI prevention globally is 46% (95% CI: 21–72%, p = 0.00), and the pooled prevalence of moderate knowledge is 27% (95% CI: 16–38%, p = 0.00), respectively.

#### Factors associated with knowledge of nurses towards SSI prevention

**Fig 8:**
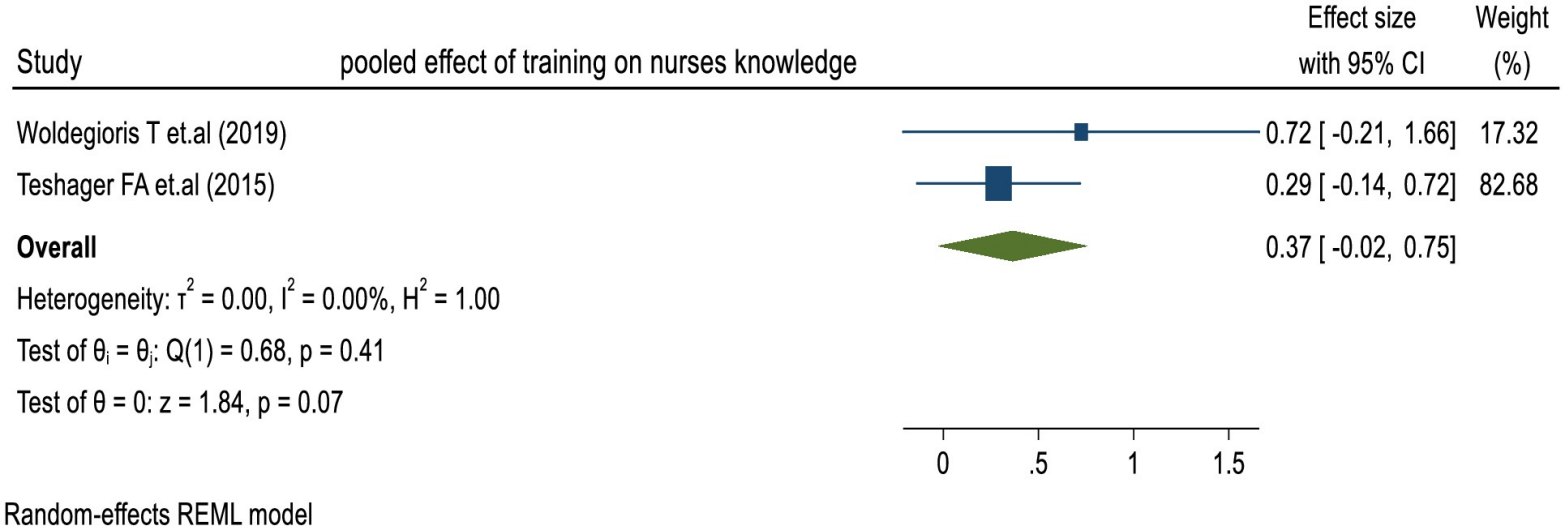
Forest plot to show effect of factors on nurses’ knowledge.

While some pooled ORs lacked significance, closer examination revealed significant associations and trends in individual studies. Service duration of more than 5 years and a history of training in infection prevention emerged as significant factors associated with nurses’ knowledge of SSI prevention in two prior studies, with adjusted odds ratios (AORs) ranging from 1.81 to 8.9 and 1.95 to 5.3, respectively [24, 25]. The random-effects model analysis of these studies showed that the pooled effect of training history on nurses’ knowledge was 0.37 (95% CI: -0.02, 0.75) (I² = 0.00%; p < 0.001) (**Fig 8**).

## Discussion

To our knowledge, this is the first meta-analysis to synthesize data on nurses’ knowledge of SSI prevention. Previous studies have primarily been limited to individual surveys and observational studies, lacking a broader synthesis of evidence. This meta-analysis provides a valuable aggregated estimate of nurses’ knowledge, overcoming the limitations of smaller, isolated studies. By pooling data, we offer a more robust understanding of the knowledge gaps and the main contributing factors that exist globally.

Our findings indicate that the pooled proportion of nurses with good knowledge of SSI prevention is 62% (95% CI: 50–74%) when assessed using a dichotomous scale. However, when knowledge is measured using a three-point Likert scale, the pooled proportion of those with good knowledge drops to 46% (95% CI: 21–72%), with an additional 27% (95% CI: 16–38%) demonstrating fair or moderate knowledge. These results are consistent with the findings of Horgan et al. [35], who reviewed the knowledge of healthcare professionals regarding SSI prevention. Although their review did not provide pooled proportions and included all healthcare providers, it similarly highlighted inadequate knowledge of SSI prevention among healthcare professionals, reinforcing the need for targeted interventions to enhance knowledge levels across the healthcare workforce.

Despite significant advancements in surgical techniques and infection control measures, the incidence and prevalence of surgical site infections (SSIs) remain alarmingly high worldwide [5]. These rates may be underestimated due to SSIs that occur after hospital discharge, often going unreported [36]. A global review of 44,814 patients undergoing elective surgeries found that SSIs occur in approximately 5% of cases, emphasizing the widespread nature of this issue [37]. In the United States, SSIs are the second most common healthcare-associated infection (HAI), significantly affecting patient outcomes and placing a strain on healthcare resources [38]. In Europe, SSIs are the third most frequently reported HAIs, accounting for 18.4% of infections according to a recent point prevalence study by the European Centre for Disease Prevention and Control (ECDC) [39].

While SSIs are largely preventable, our systematic review and meta-analysis reveal that nurses’ knowledge of SSI prevention remains suboptimal. These findings underscore the urgent need for improved preventive strategies and educational interventions to address the ongoing burden of SSIs and improve patient care outcomes globally.

The high level of heterogeneity observed among the included studies reflects the varying levels of knowledge across different regions and settings, highlighting the importance of our pooled estimates in identifying the underlying factors influencing nurses’ knowledge levels. This high heterogeneity might be related to differences in study populations: Studies could vary in terms of the demographic regions reflect differences in healthcare systems, resource availability, and cultural approaches to infection control practices. Such variations influence both knowledge and application of SSI prevention measures. Professional difference of nurses in age, years of experience, level of education, or variations in nurses’ roles such as general duty vs. specialized surgical nurses, OTR nurses can lead to differences in the exposure to and understanding of SSI prevention protocols. Nurses from different healthcare settings such as nurses from urban vs. rural hospitals, public vs. private sectors might have varying levels of exposure to SSI prevention practices [40, 41].

Differences in educational background and training, such as variations in nursing curricula, the extent of continuing professional education, and access to in-service training on infection prevention, significantly contribute to heterogeneity in systematic reviews and meta-analyses on SSI prevention. Research shows that curricula tailored to specific regional needs or healthcare contexts often vary widely, leading to differences in infection control competencies among nurses. Additionally, some healthcare systems prioritize infection prevention training more than others, influencing the consistency and depth of knowledge. For instance, infection prevention training vetted by trusted organizations, such as the CDC, tends to be more effective and widely accepted compared to locally produced programs. Customizing training to address specific professional roles further highlights the influence of educational variations on knowledge and practice disparities [42, 43].

Sample size of individual primary studies is a key contributor to high heterogeneity in meta-analyses. Evidence demonstrates that studies with small sample sizes tend to increase variability and heterogeneity. Effect sizes from smaller studies are more likely to be skewed, inflated, or deflated due to sampling error and broader confidence ranges. Furthermore, small-study effects often introduce bias as such studies may not represent the broader population due to limited data and less robust designs [44].

While this meta-analysis indicates a trend toward a positive effect of training on nurses’ knowledge of SSI prevention, the pooled odds ratio is not statistically significant. However, evidence from various sources highlights the profound impact of training on healthcare providers’ understanding of risk factors, pathophysiology, and prevention strategies for SSIs [45]. Training ensures that healthcare providers remain informed about emerging best practices, such as those recommended by the World Health Organization (WHO) and the Centers for Disease Control and Prevention (CDC), to mitigate SSIs, a significant cause of postoperative morbidity and mortality. This is particularly critical in resource-limited settings, where variations in practice are common [46]. Regular and updated training sessions enhance adherence to protocols like preoperative skin preparation, proper sterilization techniques, antibiotic prophylaxis, and postoperative care. Moreover, these sessions reinforce knowledge retention and practical application, reducing lapses in SSI prevention over time, which is essential for lowering SSI rates [47].

Healthcare providers with longer service years accumulate practical knowledge and experience over time through encounters with various scenarios, complications, and patient outcomes. This exposure allows them to develop a nuanced understanding of SSI prevention strategies and adapt these practices to specific contexts. As a result, they become more confident in implementing evidence-based practices such as proper surgical hand hygiene, sterilization techniques, and antibiotic prophylaxis. This growing confidence enhances their decision-making abilities and adherence to SSI prevention guidelines [48, 49]. The level of education plays a critical role in shaping healthcare providers’ knowledge in surgical site infection (SSI) prevention. Higher educational attainment enhances understanding of SSI risk factors and evidence-based practices, leading to improved adherence to infection control protocols. Providers with advanced education are better equipped to critically assess and apply guidelines and research findings, ensuring more effective clinical interventions towards SSI prevention [50–52].

Furthermore, the absence of heterogeneity across the included studies suggests consistent findings, which may be influenced by geographical factors, as many of the studies were conducted in Ethiopia. Unique contextual elements in this region could contribute to uniformity in results. However, further research with more diverse and comprehensive datasets is necessary to confirm the long-term effectiveness of training, years of experience, levels of education, and other pertinent variables.

These findings have significant implications for clinical practice and policy, emphasizing the urgent need for targeted educational interventions to enhance nurses’ knowledge of SSI prevention. Our results also provide a foundation for future research to explore effective strategies in diverse healthcare settings. Given the scarcity of prior meta-analyses in this area, our study serves as a critical first step. Future research should aim to investigate the factors that influence nurses’ knowledge and rigorously evaluate the impact of educational interventions across various contexts.

## Conclusion and recommendation

This systematic review and meta-analysis, is the first to synthesize data on nurses’ knowledge of SSI prevention, with only 62% demonstrating good knowledge on a dichotomous scale and just 46% on a three-point Likert scale. The significant variation in knowledge levels across different regions highlights the urgent need for targeted educational interventions to bridge these gaps globally. To enhance patient care, healthcare systems must prioritize tailored training programs and continuous professional development for nurses. Future research should focus on identifying the factors that influence nurses’ knowledge levels, with an emphasis on longitudinal and interventional studies to address significant barriers and enablers such as work environment, workload, educational resources, and personal factors effectively. Policymakers can integrate SSI prevention into standard nursing curricula by embedding international guidelines, such as those from the World Health Organization (WHO) and the Centers for Disease Control and Prevention (CDC), into course materials. This should be complemented by robust assessment tools, including practical exams, objective structured clinical examinations (OSCEs), and case-based discussions, to evaluate students’ understanding and application of SSI prevention practices. Additionally, nursing educators must be trained on the latest SSI prevention strategies to ensure effective teaching and mentorship. By integrating these elements, nursing education can equip future nurses with the competencies needed to implement best practices, significantly reducing the global burden of SSIs.

### Limitation

This review and meta-analysis included studies conducted globally, resulting in substantial to high heterogeneity, which reflects the varying levels of knowledge across different regions. This variability may be attributed to differences in tools used, sample characteristics, and regional contexts. Additionally, most of the included studies did not adequately address the factors influencing knowledge levels, limiting our ability to identify the ideal determinants that affect nurses’ knowledge of SSI prevention.

## Supporting information

S1 Table Quality appraisal of the included studies using 8 scale JBI (Joanna Briggs Institute) tool. (DOCX)

S2 Table PRISMA 2020 checklist for reporting systematic reviews and meta-analyses. (DOCX)

S3 Table Summary of identified papers and extracted information from included studies. (XLSX)

S4 Table Summary of extracted information from included studies. (XLSX)
