# Supplementary material for: Nurses’ knowledge and its determinants in surgical site infection prevention: A comprehensive systematic review and meta-analysis
Source: PLoS One. 2025 Jan 29;20(1):e0317887. doi: 10.1371/journal.pone.0317887 (PMC11778644; doi:10.1371/journal.pone.0317887)
Supplement: S1 Table — (DOCX) [file pone.0317887.s001.docx]

| No. | Critical Appraisal tools we used in JBI Systematic Reviews: The score is 1 for “Yes”, 0 for “no”, and U for “unclear”. |
| --- | --- |
|  |  |
| 1 | Were the criteria for inclusion in the sample clearly defined? |
| 2 | Were the study subjects and the setting described in detail? |
| 3 | Was the exposure measured in a valid and reliable way? |
| 4 | Were objective, standard criteria used for measurement of the condition? |
| 5 | Were confounding factors identified? |
| 6 | Were strategies to deal with confounding factors stated? |
| 7 | Were the outcomes measured in a valid and reliable way? |
| 8 | Was appropriate statistical analysis used? |
|  | Total score out of 8(%) |

Table 1s: Quality appraisal of the included studies using 8 scale JBI (Joanna Briggs Institute) tool.

| Author (Studies) | YEAR | Q1 | Q2 | Q3 | Q4 | Q5 | Q6 | Q7 | Q8 | TOTAL |
| --- | --- | --- | --- | --- | --- | --- | --- | --- | --- | --- |
| Abd Elhay HA et.al | 2016 | NO | YES | YES | YES | NO | NO | YES | YES | 5 |
| Ayamba EVE et.al | 2022 | NO | YES | YES | YES | NO | NO | YES | YES | 5 |
| Jaleta P et.al | 2021 | YES | YES | YES | YES | YES | YES | YES | YES | 8 |
| Haleema Sadia et al | 2017 | NO | YES | YES | YES | NO | NO | YES | YES | 5 |
| Shaheen SR and Hawash MAH | 2021 | YES | YES | YES | YES | YES | YES | YES | YES | 8 |
| Sham F et.al | 2021 | NO | YES | YES | YES | NO | NO | YES | YES | 6 |
| Woldegioris T et.al | 2019 | YES | YES | YES | YES | YES | YES | YES | YES | 8 |
| Teshager FA et.al | 2015 | NO | YES | YES | YES | YES | YES | YES | YES | 7 |
| KHALID N et.al | 2023 | YES | YES | YES | YES | NO | NO | YES | YES | 6 |
| Tiwari RV and Tiwari HD | 2022 | YES | YES | YES | YES | YES | YES | YES | YES | 8 |
| Hassan AH and Masror-Roudsary D | 2023 | YES | YES | YES | YES | NO | NO | YES | YES | 6 |
| Naji Msc BA et.al | 2020 | NO | YES | YES | YES | NO | NO | YES | YES | 5 |
| Patil VB et.al | 2018 | NO | YES | YES | YES | NO | NO | YES | YES | 5 |
| Asmaa Salah EL-Azab1et. al | 2023 | YES | YES | YES | YES | YES | YES | YES | YES | 8 |
| Mohammed Alsaadi and Elfeshawy R | 2024 | NO | YES | YES | YES | NO | NO | YES | YES | 5 |
| Joshi R | 2014 | NO | YES | YES | YES | NO | NO | YES | YES | 5 |
| Famakinwa T et.al | 2014 | YES | YE | YES | YES | NO | NO | YES | YES | 6 |
| Mean score | | | | | | | | | 6.24 | |

Interpretation of the score

A score of 1-3 is categorized as **Low quality**

A score of 4-6 is categorized as **Moderate quality**

A score of 7-8 is categorized as **High quality**
